# Diagnostic work-up in periprosthetic joint infections of the knee: can the albumin-to-globulin ratio be a screening tool?

**DOI:** 10.1186/s10195-025-00857-8

**Published:** 2025-07-02

**Authors:** Domenico De Mauro, Tiziana Ascione, Enrico Festa, Lucrezia Marasco, Filippo Leggieri, Sara Rosito, Matteo Innocenti, Edoardo Di Pace, Giovanni Balato

**Affiliations:** 1 Department of Public Health, Orthopedic Unit, “Federico II” University, Via S. Pansini 5, 80131 Naples, Italy; 2 Department of Orthopedics and Geriatric Sciences, Catholic University of the Sacred Heart, Largo F. Vito, 00168 Rome, Italy; 3 Department of Infectious Diseases, Service of Infectious Diseases, AORN “Dei Colli” – Via Cardarelli, AORN A. Cardarelli Hospital, Via Cardarelli, 9, 80131 Naples, Italy; 4 Department of Clinical Orthopedics, Careggi University Hospital, University of Florence, Largo G.A. Brambilla, 3, 5013450134 FlorenceFlorence, Italy

**Keywords:** PJI, Diagnostic, Albumin, Globulin, Serum markers, Total knee arthroplasty

## Abstract

**Background:**

This study aimed to assess the most appropriate thresholds for albumin-to-globulin ratio (AGR) in patients who had a suspected periprosthetic knee infection. Furthermore, the diagnostic accuracy of the proposed threshold was evaluated.

**Materials and methods:**

Between January 2020 and April 2022, patients with failed or painful knee arthroplasty who were admitted to a tertiary referral institution undergoing the standardized diagnostic protocol to identify those with a periprosthetic joint infection (PJI) were analyzed. The 2018 International Consensus Meeting (ICM) criteria were used to classify patients with PJIs and aseptic joints. Sensitivity, specificity, positive predictive value, negative predictive value, and the area under the receiver operating characteristic (ROC) curve (AUC) of AGR were calculated to define the test’s diagnostic accuracy.

**Results:**

The ROC curve showed that the optimal cutoff value of AGR was 1.43. AGR registered a sensitivity of 95% (95% CI 91–197%), a specificity of 63% (95% CI 56–69%), a positive predictive value of 75% (95% CI 69–81%), and a negative predictive value of 91% (95% CI 86–94%). Receiver operator curve analysis demonstrated an AUC of 0.85 (95% CI 0.77–0.88). Although body mass index (BMI), uremia, glutamic-oxaloacetic transaminase (GOT), international normalized ratio (INR), and alkaline phosphatase showed significant differences between the false positive cases and those cases affected by aseptic failure with AGR higher than 1.43, indicating potential confounding effects (*p* < 0.05), no parameter was found to be a significant predictor of false positives cases (*p* > 0.05).

**Conclusions:**

For its high sensitivity, AGR showed potential as a screening tool for detecting infections in PJI diagnostics.

*Level of evidence*: III.

## Introduction

Periprosthetic joint infection (PJI) is one of the most severe complications after prosthesis implantation, even considering that the diagnosis still represents a major challenge for orthopedic surgeons. Despite significant advancements in diagnosing PJIs in recent years [4, 5, 12, 28, 15, 18, 26, 31, 6, 10, 20, 39], a single test that achieves excellent diagnostic performance in detecting a joint infection is still lacking [1, 19, 35, 40, 42, 9]. Nowadays, to make a proper diagnosis, we rely on the definition of prosthetic infection proposed by the second International Consensus Meeting on PJI [28, 15], subsequently changed by the definition of PJI proposed by the European Bone and Joint Infection Society (EBJIS) [29]. These criteria include a combination of clinical and laboratory findings, some of these are evaluable in the blood, such as serum C-reactive protein (CRP) [12, 28, 31], D-dimer [1, 35, 42], and erythrocyte sedimentation rate [12, 28, 31], and others in synovial fluid, such as leukocyte esterase [7, 19, 31, 32], synovial CRP [3, 29, 38], alpha defensin [8, 24, 30, 31–38], elevated synovial fluid white blood cell count [7, 12, 13, 17, 30], and the polymorphonuclear percentage [7, 12, 13, 17]. None of these alone achieve a high diagnostic accuracy [15, 41]. For this reason, new cheap, fast, and minimally invasive biomarkers are recently proposed. Serum albumin (Alb) and globulin (Glb) and their ratio (AGR) have gained considerable interest in literature.

Albumin, a major protein synthesized in the liver, is crucial in maintaining oncotic pressure and transporting various substances throughout the body [22]. Globulins, conversely, are a group of proteins involved in immune responses and also produced in the liver [25]. The cost of evaluating Alb and Glb levels is relatively low, making it a cost-effective option in clinical settings, thus it has been investigated from the oncological to the mental health field [25, 27, 33]. The quantitative assessment of serum Alb and Glb levels, and their ratio, has shown interesting evidence in diagnosing PJI in recent investigations [13, 16, 43, 44]. A systematic review of third-level evidence papers has recently been conducted to assess the effectiveness of AGR in diagnosing PJIs. Although the results are promising, the total number of cases reviewed remains relatively small, especially for periprosthetic knee infections.

This study therefore aimed to address two questions: (1) What are the most appropriate thresholds for AGR in patients with painful total knee arthroplasty (TKA)? and (2) What is the diagnostic accuracy of our proposed thresholds in the diagnostic work-up?

## Materials and methods

Consecutive patients with failed or painful knee arthroplasty who were admitted to a tertiary referral institution between January 2020 and April 2022 to undergo the standardized diagnostic protocol to identify those with a PJI were analyzed retrospectively.

All investigations were conducted in conformity with the ethical standards of the institutional and national research committee and with the 1964 Helsinki Declaration and its later amendments. The patients provided informed consent before they were included in the study. The standard workup included clinical evaluation, CRP, erythrocyte sedimentation rate (ESR), and D-dimer, joint aspiration for white blood cell (WBC) count, percentage of polymorphonuclear leukocytes (PMN) count, and synovial fluid cultures. The diagnosis of chronic infection (> 90 days after the index procedure) was made according to the Second International Consensus Meeting (ICM) definition of PJI [32]. Exclusion criteria were: chronic inflammatory joint diseases (e.g., rheumatoid arthritis, psoriatic arthritis); acute (< 90 days after the index procedure) and late hematogenous infections (symptoms of less than 3 weeks duration); an inadequate amount of synovial fluid (≤ 10 mL) for culture, WBC, and PMN (neutrophil) percentage determinations; and insufficient serum marker data. Patients withdrew any antibiotic treatment at least 2 weeks before the diagnostic procedure.

Albumin and globulin levels and their ratio (AGR) were evaluated for all the subjects included in the study. Sex, age, body mass index (BMI), and Charlson Comorbidity Index (CCI) were also recorded from the hospital’s electronic registry.

For each patient, an additional workup on venous blood samples was performed to assess coagulation-related markers, including INR, renal function markers (creatinine and uremia), and liver function, such as alkaline phosphatase (ALP), glutamic-oxaloacetic transaminase (GOT), and glutamic pyruvic transaminase (GPT).

### Statistical analysis

Median and interquartile range were used for non-normal distributed continuous variables, and mean and standard deviation for normal distributed continuous variables. For categorical variables, frequencies and percentages were used and compared using the Chi-squared test or Fisher’s exact probability test.

Receiver operating characteristic (ROC) curves, which depict relationships between true-positive results (sensitivity) and false-negative results (1−specificity), were constructed for the AGR. The parameters’ sensitivities, specificities, positive predictive values (PPVs), and negative predictive values (NPVs) were calculated using 2 × 2 contingency tables. The area under the ROC curve (AUC) was assessed to evaluate the diagnostic parameter accuracy better. An AUC of 1 indicates 100% sensitivity and 100% specificity, while an AUC < 0.5 indicates a less useful diagnostic test. An internal validation method through a bootstrapping method was used to obtain the optimal cutoff value for AGR. A total of 1000 bootstrap samples from the 237 patients were drawn with replacements in the original data. The advantage of this method is that the bootstrap-based ROC curves are much more stable than those of holdout or cross-validation analysis, indicating a more stable ROC analysis. This is performed by considering a misclassification cost function (to be minimized) to assess the discriminatory ability of a cutoff point that relied on the elements of the 2 × 2 confusion matrix, that is, true positives (TP), false positives (FP), true negatives (TN), and false negatives (FN), which is cost FP_3 FP + cost FN 3 FN. We assumed that the cost of false-negative results is three times higher than false-positive results. Furthermore, Spearman correlation was used to identify relationships between AGR and potential confounders among false positives. The variables that reached a statistical difference between patients with false-positive and true-negative results were then evaluated in an age-adjusted multivariate logistic regression model. A value of p < 0.05 indicated statistical significance. The R statistical software environment (IBM SPSS software, version 21.0.0.1, IBM Corp) was used to construct the databases and conduct the statistical analyses.

## Results

### Patient characteristics

**Fig. 1:**
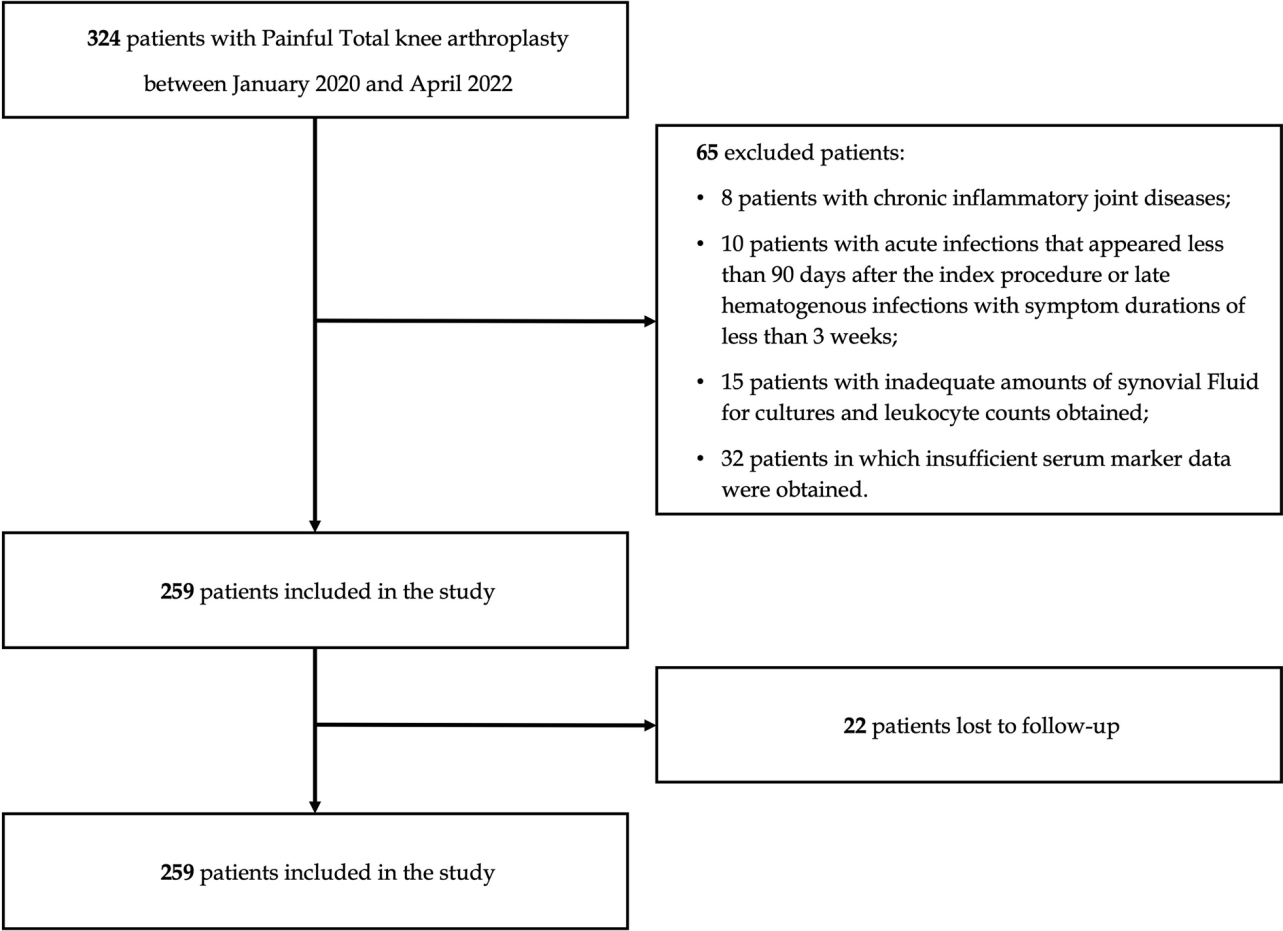
Flowchart of patient selection from the local database

Of the 324 patients initially evaluated, 237 were included in the final analysis on the basis of the predefined exclusion criteria (Fig. 1). The median age was 72 years (range 48–91 years), and the majority were female (58%). According to the 2018 International Consensus Meeting criteria, 129 cases were diagnosed with periprosthetic joint infection (PJI), with coagulase-negative *Staphylococci* being the most frequently isolated organisms. Table 1 presents the demographic and clinical characteristics of patients with PJI and those with aseptic failure. No significant differences were observed between the two groups regarding age, sex distribution, or Charlson Comorbidity Index. In contrast, patients with PJI exhibited significantly higher values of body mass index (BMI), erythrocyte sedimentation rate (ESR), C-reactive protein (CRP), synovial fluid white blood cell (WBC) count, and polymorphonuclear cell (PMN) percentage (all *p* < 0.001), consistent with an inflammatory response. Notably, the albumin-to-globulin ratio (AGR) was significantly lower in the infection group (median 1.1, IQR 0.93–1.35) compared with the aseptic group (median 1.5, IQR 1.32–1.7; *p* < 0.001), supporting its potential role as an adjunctive biomarker in the diagnostic assessment of PJI.

**Table 1:** Characteristics of patients with or without periprosthetic knee infection

	PJI Group *N* = 129	Aseptic group *N* = 108	*p*-Value
Age (years)	70 (IQR 63–74.5)	71 (IQR 65–76.25)	0.373
Male sex (no. [%])	52 (40%)	46 (43%)	0.142
BMI	29.3 (IQR 26.3–34.1)	27.4 (IQR 25.45–30)	< 0.001
CCI	3 (IQR 2–4)	3 (IQR 2–4)	0.320
ESR mm/h	35 (IQR 18–57)	22 (IQR 12.3–30)	< 0.001
CRP mg/L	16.7 (IQR 4.3–41.5)	3.2 (IQR 1.03–5.63)	< 0.001
SF – WBC count (cells/uL)	8511.5 (IQR 623.5–21,435)	450 (IQR 191.5–1003.25)	< 0.001
SF – PMN	91 (IQR 77–94.9)	37.5 (IQR 24.74–54.25)	< 0.001
AGR	1.1 (IQR 0.93–1.35)	1.5 (IQR 1.32–1.7)	< 0.001

*CRP* C-reactive protein, *ESR* erythrocyte sedimentation rate, *IQR*  interquantile range, *PJI* periprosthetic joint infection, *SF – WBC* synovial fluid white blood cell, *SF – PMN* synovial polymorphonuclear; *AGR* albumin-to-globulin ratio, *CCI* Charlson Comorbidity Index, *BMI* body mass index

### Predictive value of proposed cutoffs for AGR levels

**Fig. 2:**
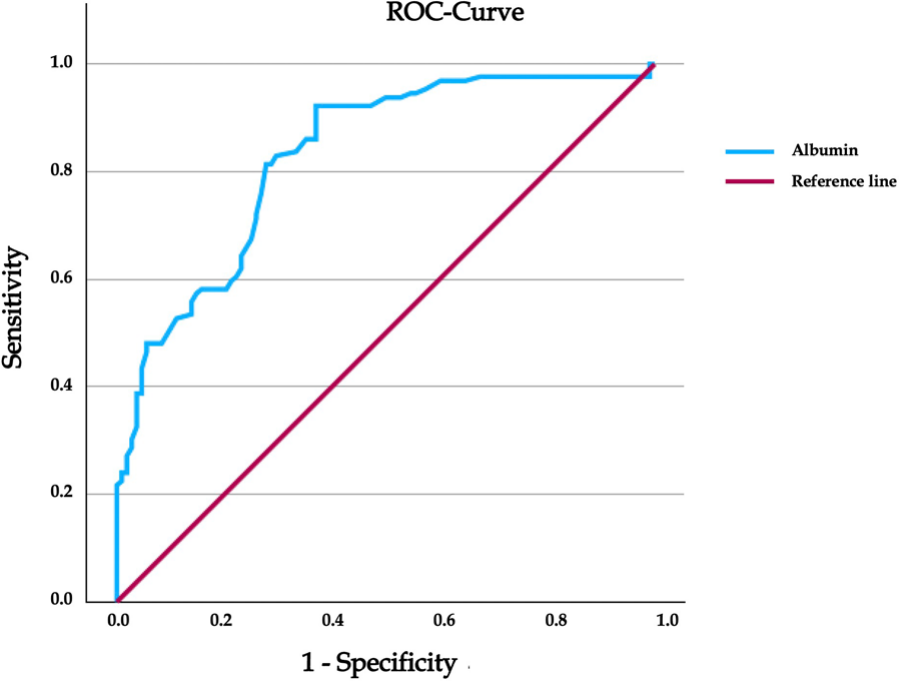
Graph shows receiver operating characteristic (ROC) curve and area under curve (AUC) of AGR

ROC curve analysis, used to identify the best threshold value, showed that an AGR of 1.43 (proposed threshold) represents the best cutoff value (Fig. 2). The AGR evaluation correctly identified 122 of 129 patients with PJIs and 68 of 108 patients with aseptic failures of the implant. We reported only 7 false-negative and 40 false positive results with a sensitivity of 95% (95% CI 89–98%), a specificity of 63% (95% CI 53–72%), a positive predictive value of 75% (95% CI 70−80%), and a negative predictive value of 91% (95% CI 82–95%). ROC analysis demonstrated an AUC of 0.85 (95% CI 0.77–0.88).

### False-positive cases subgroup analysis

A Mann–Whitney *U* test highlighted significant differences between the true-negative and false-positive cases for several biomarkers. BMI, uremia, GOT, and alkaline phosphatase showed significant differences, indicating potential confounding effects (*p* < 0.05). Spearman correlations showed significant negative correlations were observed with ALP (*p* < 0.001), BMI (*p* = 0.036), uremia (*p* = 0.010), and ALT (*p* = 0.041). A significant correlation was observed also for INR (*ρ* = 0.329, *p* = 0.006). Univariate logistic regression was performed to assess the eventual biochemical and clinical parameters that affect the false-positive results, which are expressed in Table 2. None of the parameters reached statistical significance, failing to predict false positives (*p* > 0.05).

**Table 2:** Univariate age adjusted logistic regression analysis for potential confounders predicting false-positive cases

Variable	B	S.E.	Wald	df	*p*-Value	OR
BMI	−0.058	0.051	1.264	1	0.261	0.944
Creatinine	0.053	0.11	0.232	1	0.63	1.054
Azotemia	−0.018	0.016	1.182	1	0.277	0.982
GOT	0.021	0.021	0.973	1	0.324	1.021
GPT	−0.005	0.018	0.063	1	0.802	0.995
INR	−0.484	0.602	0.647	1	0.421	0.616
Alkaline phosphatase	0.0	0.011	0.0	1	0.991	1.0

*BMI* body mass index, *GPT* glutamate pyruvate transaminase, *GOT* glutamic-oxaloacetic transaminase, *INR* international normalized ratio

## Discussion

The most important finding of this study is represented by the evaluation of AGR in detecting knee periprosthetic joint infections. The evaluation of AGR in patients affected by PJI and aseptic failure is well described in literature. The rationale is supported by the association between globulin and AGR values, and patients with poor immunity status [13]. Consequently, there is a higher risk of complications, including PJI [13, 16, 43]. In addition, globulin and AGR cost-effectiveness make these values easily accessible, and they could implement actual diagnostic work-up, to predict and reduce severe complications, particularly in patients with higher globulin or low AGR. A meta-analysis recently reported a pooled sensitivity and a specificity of 75%, highlighting AGR’s good diagnostic accuracy as a screening tool in painful total joint arthroplasty [13]. In this paper, lower ratio values were found in patients affected by prosthetic infection rather than patients with aseptic failures, with a statistically significant difference. These results are in line with those reported by Choe et al. [14], Li et al. [27], Wu et al. [44], Dong et al. [16], Wang et al. [43], and Jiao et al. [21], which confirmed the differences between aseptic and septic patients, thus hypothesizing the role of this parameter in the diagnostic approach to PJIs. With 7 false-negative results and 40 false-positive results, the sensitivity, specificity, positive predictive value, and negative predictive value were 95%, 63%, 75%, and 91%, respectively. In addition, we identified 1.43 as the optimal AGR cutoff point to diagnose PJI in TKA patients. The proposed cutoff is higher than those reported in previous studies. Conversely, the achieved diagnostic accuracy is lower than those reported by previous studies, which consider patients affected by periprosthetic knee infections. Indeed, Li et al. [27] and Dong et al. [16] reported an AUC higher than 85%, indicating a high performance level of this parameter in diagnosing knee infections. The patients who had false‑negative AGR results were nevertheless classified as infected because, according to the 2018 ICM diagnostic criteria [32], presented two positive cultures or a ≥ 6 score. Negative AGR results in patients with confirmed PJI could be explained by the presence of the sinus tract, low-grade infection, and previous antibiotic administration that reduces the local accumulation of pathogens, resulting in a weaker immune response [24, 45].

Dampened inflammation could also explain the lack of reduction in albumin serum values, and the lack of increase in globulin levels. Conversely, in the current cohort, 40 false-positive results were reported. Previous studies reported the potential cause of false-positive results in serum AGR values [13] may be due to the common production of albumin and globulin in the liver. Thus, a pathological change in AGR values can be determined by cirrhosis, liver malignancies, and other inflammatory diseases. A previous study demonstrated a strong association between high serum globulin levels and the extent of hepatic fibrosis in patients with chronic hepatitis B infection [1]. Similarly, Schmilovitz-Weiss et al. reported that high serum globulin levels could serve as a marker to predict the extent of hepatic fibrosis in patients with post-transplant recurrent hepatitis C infection [11].

Moreover, a low AGR is a valuable marker for predicting poor prognosis in patients with cancer [2]. Other factors can influence specificity, including ankylosing spondylitis, rheumatoid arthritis, tuberculosis, and hematopoietic failure, potentially limiting the value of these serological indices in real-world clinical contexts. A subgroup analysis was performed to explore the role of other markers and clinical conditions, including inflammatory markers, kidney and liver function markers, and BMI, as potential contributors to false-positive results. Unlike BMI: uraemia, GOT, and alkaline phosphatase showed significant differences, indicating potential confounding effects, none of the parameters reached statistical significance, failing to predict false positives.

The diagnostic power of AGR (high sensitivity 95% and low specificity 63%), suggests that it may serve best as a screening tool rather than a stand-alone confirmatory test. The low rate of false negatives underscores its value in ruling out infection. This is particularly relevant in preoperative evaluations where early identification of potential infection can alter surgical planning and reduce the risk of adverse outcomes. However, the higher proportion of false positives introduces a risk of over-investigation or overtreatment. These patients may undergo unnecessary joint aspirations, biopsies, or even unwarranted revision surgeries if AGR results are misinterpreted in isolation. Therefore, its clinical use should prioritize ruling out infection rather than confirming it. In AGR-positive cases, clinicians should interpret the result in the context of a comprehensive diagnostic work-up—including synovial fluid analysis, cultures, and established scoring systems—rather than relying on AGR alone to confirm infection.

Compared with classical markers such as CRP, ESR, and D-dimer, AGR shows a favorable sensitivity profile but lower specificity. For instance, CRP has high sensitivity but may be elevated in numerous non-infective conditions, while D-dimer is known for its high false-positive rate, especially in elderly or comorbid patients. Synovial biomarkers such as alpha-defensin and leukocyte esterase offer superior specificity, but are more expensive, technically demanding, or less widely available in all clinical settings. Moreover, AGR presents advantages in terms of cost, speed, and availability, since albumin and globulin levels are routinely tested in standard biochemistry panels.

This study’s findings present several limitations. First, a group of patients with periprosthetic knee infection without chronic inflammatory joint disease was investigated. This made the study population homogeneous and reduced some relevant biases, but also prevented the possible cutoff application to the entire population of patients affected by PJI, including those with chronic inflammatory diseases, or with other failed joint prostheses. Second, some cofactors that could influence the diagnostic accuracy of AGR, such as patient comorbidities, type of infection, and bacterial virulence, should be more widely investigated. Although the sample was well-balanced, its small size precluded assessing the cutoff value proposed in these selected subpopulations. Further investigations on the role of these factors should be considered, enrolling a significantly higher number of cases.

To our knowledge, this is the largest series in the literature on painful TKA prostheses. Furthermore, this study adopts a bootstrap technique as an internal validation to quantify any optimism in the predictive performance that leads to an increase in the variance, thus obtaining a more realistic simulation of the real-world experiment from which our dataset was obtained. In addition, it addressed the choice of an “optimal” cutoff point, with attention given to misclassification cost to evaluate the classifier’s performance.

## Conclusions

The AGR threshold for diagnosing knee PJI showed high sensitivity and performed well in true-positive case identification but with low specificity, determining high rates of false positives. However, even if the current study findings support using a high-sensitivity threshold for screening purposes, ensuring that most cases of PJI are detected, more specific diagnostic tools should be performed in AGR-positive cases.

## Data Availability

The datasets generated and/or analyzed during the current study are not publicly available due to human subjects research considerations regarding the sensitivity of information. Deidentified data can be available from the corresponding author upon reasonable request.
